# Perspectives on Specific Carbohydrate Diet Education from Inflammatory Bowel Disease Patients and Caregivers: A Needs Assessment

**DOI:** 10.1097/PG9.0000000000000222

**Published:** 2022-07-25

**Authors:** Nancy Rivera, Kaylie Nguyen, Venus Kalami, Rebecca Blankenburg, Ann Ming Yeh

**Affiliations:** From the * Division of Pediatric Hospital Medicine, Kaiser Permanente Santa Clara Medical Center, Santa Clara, CA; † Division of Pediatric Gastroenterology, Hepatology, and Nutrition, Stanford Children’s Health, Lucile Packard Children’s Hospital, Palo Alto, CA; ‡ Division of Pediatric Gastroenterology, Hepatology, and Nutrition, Stanford Children’s Health, Lucile Packard Children’s Hospital, Palo Alto, CA; § Division of Pediatric Hospital Medicine, Department of Pediatrics, Stanford School of Medicine, Palo Alto, CA; ∥ Division of Pediatric Gastroenterology, Hepatology, and Nutrition, Department of Pediatrics, Stanford School of Medicine, Palo Alto, CA.

## Abstract

The specific carbohydrate diet (SCD) has potential health benefits for inflammatory bowel disease (IBD); however, adherence is challenging. Through an online needs assessment survey, this study explored the perspectives of patients and caregivers using the SCD to manage IBD to determine barriers, knowledge gaps, and desired areas for further learning about the SCD. Inclusion criteria included patients with IBD or their caregivers who had experience with the SCD. Of the 208 participants, 87% of participants were female with a mean age of 46 years. Fifty-seven percent had never received SCD training before starting the diet. Participants favored more education on several topics within the SCD and identified one-on-one sessions as the preferred learning modality. Barriers identified were initial steep learning curve, time commitment, and a desire for more support from healthcare professionals. This needs assessment survey highlights the gaps in educational priorities for patients on the SCD.

**What Is Known**

- Diet-based approaches, such as the specific carbohydrate diet (SCD), may be effective therapies for patients with inflammatory bowel disease (IBD).
- The degree of education that patients receive before starting and while on the SCD is highly variable.

**What Is New**

- Patients and caregivers are motivated to try nonpharmacologic approaches to treatment.
- Patients and caregivers identified having minimal support and education from healthcare professionals as a barrier to starting the SCD.
- This study describes current educational resources being used to learn the SCD and educational content topics and curricular format that individuals are interested in.

**Translational Impact**

- The study has identified preferred educational content and formats to learn more about the SCD. These results can be leveraged to create a future curriculum on the SCD.
- Increasing educational content on the SCD to individuals, caregivers, and medical providers has the potential to improve adherence and sustainability on the SCD.

## INTRODUCTION

Research advances have improved the understanding of the dynamic relationship between diet and inflammatory bowel disease (IBD), including diet’s impact on the pathogenesis and treatment of IBD. Diet-based approaches, such as exclusive enteral nutrition and various exclusion diets, have been studied and found to be effective treatments in patients with IBD (1).

The specific carbohydrate diet (SCD) is an exclusion diet studied for its potential to induce and maintain remission in certain patients with IBD. Though several studies highlight clinical improvement in those with Crohn’s disease, some research suggests improvement for patients with ulcerative colitis as well (2–6). Studies have reported varying responses to the SCD, ranging from linear growth acceleration, decreased gastrointestinal symptoms, and inflammation, to disease remission in pediatric patients with IBD (5–8). A prospective uncontrolled trial of the SCD in patients with mild to moderate IBD found decreased disease activity scores and inflammatory markers in some of the patients, with potentially beneficial shifts in the fecal microbiome after 12 weeks on the diet (8). A randomized controlled trial found no significant difference in achieving clinical remission after 6 weeks when comparing the SCD and the Mediterranean diet in adults with Crohn’s disease (9). The PRODUCE (Personalized Research on Diet in Ulcerative Colitis and Crohn’s Disease) study is a multi-institution randomized controlled trial that is currently being conducted and aims to compare pediatric patient outcomes when comparing strict SCD versus modified SCD (10). The SCD focuses on whole, unprocessed foods, including fruits, most vegetables, nuts and seeds, various animal proteins, aged cheeses, lactose-free SCD yogurt, butter, and ghee, whereas excluding food additives, grains, most starches, and refined sugars.

Although the SCD may be an effective therapy for IBD, long-term adherence is challenging due to several barriers, such as dietary restrictions, perceived lack of variety, taste fatigue, psychosocial impacts, financial burden, time commitment, and inconsistent clinical responses. Although excellent online resources exist (eg, through the www.nimbal.org, www.NTforIBD.org, and other websites), the amount of diet education that patients receive at SCD initiation is highly variable. This study aims to explore the perspectives of patients and caregivers, using the SCD to manage IBD, to determine barriers, knowledge gaps, and desired areas for further learning.

## METHODS

A needs assessment survey was developed using the clinical experience of a team of pediatric healthcare professionals (pediatrician, pediatric gastroenterologist, nurse practitioner, dietitian nutritionist, and educators). The survey assessed characteristics of the participant completing the survey and the individual with IBD, prior SCD education, and interest in future SCD education. Demographic questions were adapted from a survey developed by Suskind et al (5). The survey was pilot tested by a group of healthcare professionals and distributed using Qualtrics Software.

In June 2020, the needs assessment survey link was shared via an internal secure patient listserv, the Facebook *SCD Families* group (https://www.facebook.com/groups/SCDFamilies), and www.NTforIBD.org. The needs assessment survey screener included a project summary, consent form, screening questions, and survey questions. The survey was in English, and all participants read and wrote in English. Inclusion criteria included patients with IBD or their caregivers who have experience with the SCD.

### Statistical Analysis

Descriptive and frequency statistics were used to analyze the quantitative data with Excel. Content analysis and grounded theory were used to analyze themes within the qualitative data, with two investigators (NR and KN) reviewing the data independently and assigning codes. The investigators compared their codes line by line, until consensus was achieved. Categories and themes were then developed. Additional investigators (AMY and VK) helped reconcile any conflicts. Frequency counts were determined by the number of times a code was mentioned.

The study was approved by the Institutional Review Board (IRB) of Stanford University (Protocol Number: 55032).

## RESULTS

### Participant Demographics

A total of 208 participants completed the survey (Table 1). Participants who completed the survey were 87% female with an average age of 46 years (range 13–82 years old); 57% were the caregivers of a child with IBD, 40% were individuals with IBD, and 3% were spouses/partners of the individual with IBD. Most had a college or a Masters/Doctorate Degree, 47% and 41%, respectively. Ninety-six percent lived in the United States, and 4% lived internationally.

**TABLE 1.** Demographics of participants and individuals with IBD following SCD

Demographic information of participant		n = 208	
Gender, female	180 (87%)		
Average age ± SD	46.4 ± 12.9 years		
Residence	USA 199 (96%)		
Highest level of education	n (%)	How is the participant related to the individual with SCD?	n (%)
High school	18 (9%)	My Child	118(57%)
College/University Degree	97 (46%)	Self	83 (40%)
Masters/Doctorate	85 (41%)	Spouse/Partner	7 (3%)
Other	8 (4%)		
**Demographic information of individual with IBD n (%)**		**n (%)**	
Type of IBD		Area of IBD disease at diagnosis	
Crohn’s disease	135 (65%)	Esophageal and stomach	57 (27%)
Ulcerative colitis	49 (24%)	Small bowel	122 (59%)
Indeterminate colitis	7 (3%)	Large bowel	145 (70%)
Unsure	17 (8%)	Extra intestinal	41 (20%)
Age of IBD diagnosis		Ever used medications to treat IBD?	
0–10 y old	59 (28%)	Yes	166 (80%)
11–17 y old	76 (37%)	No	40 (19%)
18–35 y old	44 (21%)	Unsure	2 (1%)
>36 y old	29 (14%)		
Currently following SCD?		For those currently following SCD, to what degree is SCD currently being followed?	
Yes	191 (92%)	Modify <10% of the time	136 (71%)
No	17 (8%)	Modify 10–25% of the time	49 (26%)
		Modify >50% of the time	6 (3%)
For those currently following strict SCD, for how long has strict SCD been followed?	n = 136	For those not currently following SCD, how long was SCD followed before stopping?	n = 17
<6 mo	n = 21 (15%)	<1 y	8 (47%)
Between 6 mo and 1 y	n = 19 (15%)	>1 y	9 (53%)
1–2 y	n = 22 (16%)		
>2 y	n = 74 (54%)		

Sixty-five percent of individuals with IBD had an initial IBD diagnosis at or under the age of 17 years (Table 1). The majority (65%) were diagnosed with Crohn’s disease, whereas 24% had ulcerative colitis. Of those with IBD, areas affected by IBD included: 11% esophagus, 16% stomach, 59% small bowel, 70% large bowel, and 20% had extraintestinal manifestations. Eighty percent of individuals with IBD had previously or were currently using medications to treat their IBD. The top 3 medications used were aminosalicylates (53%), corticosteroids (52%), and biologics (36%).

### Current Practices and Perspectives on the Specific Carbohydrate Diet

Ninety-two percent of individuals were following the SCD (n = 191), whereas 8% (n = 17) had discontinued the SCD at the time of the survey (Table 1). Of those individuals following the SCD, 71% were modifying the SCD <10% of the time; 26% were modifying the SCD 10% to 25% of the time; and 3% were modifying the SCD >50% of the time. Of those, modifying the SCD <10% of the time, 70% had been following the SCD for >1 year.

Participants reported various motivations for initiating the SCD including avoidance of medications (60%), utilizing an adjunct to medication therapy (32%), incorporating an integrative medicine treatment approach (28%), and following recommendations provided by a medical professional (16%) or by a family member/friend (12%). The top 3 reasons for discontinuation of the SCD included: the lack of IBD symptom improvement (29%), limited SCD support (24%), and restrictive food options (24%).

### Learning About the SCD

The top 3 resources used to initially learn about the SCD were the internet (48%), a healthcare provider (23%), and a book or a magazine (9%). Participants identified *Breaking the Vicious Cycle* (11) as the most useful resource (25%), followed by the SCD Families Facebook Group (23%) and other Facebook support groups (20%). Regarding SCD education, 57% of participants had never received SCD training before starting the diet, 21% received a session on SCD with a registered dietitian, and 13% with a medical provider.

Seventy-nine percent of participants were interested in a virtual SCD educational session, whereas 57% were interested in attending in-person at their local hospital. One-on-one sessions with health professionals were identified as the preferred learning modality, followed by teaching kitchens, online educational videos, group sessions, and newsletters in ranking order by frequency counts.

Regarding educational content, participants favored more education on the following topics: SCD resources to use at home, a beginner’s guide to the introductory elements of SCD, and in-depth explanations of ingredients allowed, in ranking order. Desired areas for further education were expressed: liberalization of the SCD (22%), science and clinical research behind the SCD (17%), illegal versus legal foods (8%), traveling and eating out (6%), and nutritional adequacy and supplements (5%).

Barriers identified with the initiation of the SCD included the initial steep learning curve and time commitment with meal preparation, in addition to a desire for more support and education from healthcare professionals and guidance on stages of the SCD (Table 2).

**TABLE 2.** Barriers identified by participants surrounding implementing the SCD

Theme	Associated quotes
Initial steep learning curve	“That even though it is very daunting in the beginning it is absolutely doable!” “That there are no shortcuts. The work involved does eventually become streamlined and easier!” “It was a total new way of thinking. Huge learning curve initially.”
More support and education fromhealth care professionals	“I wish our doctor mentioned it and that I didn’t have to search for an alternative to medication. I wish more doctors were on board with nutrition-based healing.” “It would have been wonderful to have had a tutorial program of some sort…I read as much as I could for a month or so, accumulated supplies and ingredients, and went for it.” “I wish I started much earlier in my IBD disease course. This should be part of the conversation along with access to help, insurance, money and resources.”
Time commitment involved with meal preparations	“Just that it’s a lot of work on the person doing the cooking, me for instance” “Batch cooking is key, hiring help to prepare or cook if possible. It’s so much cooking at first, it’s discouraging, but we’ve found success.” “You have to devote much time to cooking for the SCD, but it is 100 percent worth it when you can be in total remission without drugs.”
Guidance on staging	“Staging. I like there is no set amount of time but need more info of when, which food and how.” “I wish I would have known how important it is to start at the intro, I introduced food too quickly and had to restart.” “How to introduce for max result not max retention. Wish we had started even more strict and added as results were seen. Rather than just jumping into all legal foods at once.”

## DISCUSSION

This survey explores motivations surrounding SCD initiation, knowledge gaps and barriers, and desired areas for further learning among patients with IBD and caregivers. We found that participants identified the desire to avoid medication therapies, whereas also trying nonpharmacologic approaches to treatment as motivating factors. Patients and caregivers strongly called for medical providers to be familiar with all possible treatments for IBD at diagnosis, including nutritional therapy when appropriate, whereas also offering ongoing nutrition education and support given the steep learning curve pertaining to the diet. The strong desire for additional support on the SCD indicates that more consideration should be placed on nutrition education by the medical team, when appropriate.

The study identified educational resources currently being used by many individuals to learn the SCD and described educational content and format individuals will use to learn the SCD. The high utilization of internet resources and low attendance of professional educational sessions suggest that many patients are starting the SCD without formal guidance and support. Given that the SCD is a whole foods dietary intervention that requires significant food preparation and nutritional literacy, increasing available educational guidance that highlights one-on-one counseling, support groups, or interactive sessions for individuals, caregivers, and medical providers may improve adherence and sustainability on the SCD and decrease barriers to starting SCD.

The study has some limitations. Social desirability bias exists with any survey and was mitigated through making the survey anonymous. Since most respondents were recruited through online platforms, a selection bias is possible. Although this may limit generalizability, this survey may reflect individuals who are more committed to explore nutritional therapies such as the SCD. Additionally, the survey was not validated as a tool to assess motivations. This survey was conducted in English, and future studies should explore perspectives from non-English speaking patients and caregivers.

Patients and caregivers of individuals with IBD express a strong interest in education and support as they consider, initiate, and continue the SCD. The results of this study will be used to guide creation of an educational SCD curriculum for the public. Future studies should explore whether patient educational sessions improve adherence and longevity with the SCD and ultimately impact IBD outcomes. Future interventions can include increased nutritional education to medical teams that are not as familiar with nonpharmacologic approaches. Other premises for future studies about SCD education may explore whether more thorough education and support may reduce the incidence of unintended consequences associated with restrictive diets such as eating disorders.

## ACKNOWLEDGMENTS

We would like to thank Tali Guday, moderator and owner of SCD Families Facebook page and NTforIBD.org. In addition, we are grateful for the Stanford Pediatrics Residency Grant for helping fund this study.
